# Association of PvuII and XbaI polymorphisms on estrogen receptor alpha (ESR1) gene to changes into serum lipid profile of post-menopausal women: Effects of aging, body mass index and breast cancer incidence

**DOI:** 10.1371/journal.pone.0169266

**Published:** 2017-02-15

**Authors:** Neuza Felix Gomes-Rochette, Letícia Soncini Souza, Bruno Otoni Tommasi, Diego França Pedrosa, Sérgio Ragi Eis, Irani do Carmo Francischetto Fin, Fernando Luiz Herkenhoff Vieira, Jones Bernardes Graceli, Letícia Batista Azevedo Rangel, Ian Victor Silva

**Affiliations:** 1 Aging Cell Biology Laboratory, Morphology Department, Centro de Ciências da Saúde, Universidade Federal do Espírito Santo, Vitória, Espírito Santo, Brazil; 2 Programa de Pós-Graduação em Biotecnologia/RENORBIO, Centro de Ciências da Saúde, Universidade Federal do Espírito Santo, Vitória, Espírito Santo, Brazil; 3 Instituto Tommasi de Pesquisa e Desenvolvimento, Vila Velha, Espírito Santo, Brazil; 4 Centro de Diagnóstico e Pesquisa da Osteoporose do Espírito Santo–CEDOES, Vitória, Espírito Santo, Brazil; 5 Laboratory of Endocrinology and Cellular Toxicology, Programa de Pós-Graduação em Ciências Fisiológicas, Centro de Ciências da Saúde, Universidade Federal do Espírito Santo, Vitória, Espírito Santo, Brazil; 6 Laboratory of Cellular and Molecular Biology of Human Cancer, Pharmaceutical Sciences Department, Centro de Ciências da Saúde, Universidade Federal do Espírito Santo, Vitória, Espírito Santo, Brazil; Universita degli Studi di Padova, ITALY

## Abstract

Estrogen is a steroidal hormone involved in several physiological functions in the female body including regulation of serum lipid metabolism and breast cancer (BC). Estrogen actions on serum lipids mostly occur through its binding to intracellular Estrogen Receptor alpha (ERalpha) isoform, expressed in most of tissues. This gene (ESR1) exhibit many polymorphic sites (SNPs) located either on translated and non-translated regions that regulate ERalpha protein expression and function. This paper aimed to investigate the association of two intronic SNPs of ESR1 gene, namely c454-397T>C (PvuII) and c454-351A>G (XbaI) to alterations in serum levels of total cholesterol (T-chol), total lipid (TL), low density lipoprotein cholesterol (LDL), high density lipoprotein (HDL), and triglycerides (TG) in a *cohort* of post-menopausal women. In addition, we aimed to associate presence of these SNPs to development of BC along 5 years period. To do so, a group of healthy 499, highly miscigenated, post-menopausal Brazilian women, were carried using PCR-FRLP technique and further confirmed by automatic sequence analysis as well followed through 5 years for BC incidence. Measurements of serum lipid profile by standard commercial methods were carried individually whereas Dual Energy X-ray Absorciometry (DXA) measured Body Mass Indexes (BMI), Fat Mass (FM), Lean Body Mass (LBM), and Body Water Content (BWC). No effects of PvuII SNP on ESR1 gene were observed on patient´s serum T-chol, TL, LDL, HDL, and TG. However, c454-397T>C PvuII SNP is associated to lower body fat and higher levels of lean mass and body water and lower incidence of BC. On the other hand, statistically significant effect of XbaI c454-351A>G SNP on serum TG and TL levels. Patients homozygous for X allele were followed up from 2010–2015. They showed higher incidence of breast cancer (BC) when compared to either heterozygous and any P allele combination. Moreover, the increasing of TG and TL serum concentrations associated to SNP XbaI c454-351A>G on ESR1 gene is enhanced in both obese (higher BMI) and elder women. Taken together, these results suggested that XbaI c454-351A>G SNP is associated to changes in lipid profile, particularly in serum TG and TL, in this cohort of post-menopausal woman. Also, this paper shows another link between obesity and BC corroborating the current thesis that both diseases are interlinked.

## Introduction

It is well known that majority of actions of estrogen are due to its binding to intracellular estrogen receptors (ESR) which regulated gene expression. There are two well characterized intracellular isoforms of ESR, encoded by separate genes in humans [1,2]. The first known ESR1 gene (NCBI ID: +133430) located onto chromosome 6 codes the Estrogen Receptor alpha (ERalpha) protein, abundantly expressed in liver, adipose tissue, breast, and cardiovascular system [1,3]. There has been demonstrated that activated ERalpha regulates the hepatic expression of several other genes involved in lipoproteins metabolism, resulting in a raise in serum high density lipoprotein (HDL) cholesterol and triglycerides concentrations whereas decreasing serum low density lipoprotein (LDL) cholesterol and lipoprotein A II concentrations [3]. It has been shown that lack of female sex steroids hormones observed after menopause–particularly estrogen–is may lead to many diseases, such as breast cancer, osteoporosis, and increased risk of metabolic syndrome [4–7]. Taken together, the ovarian failure that characterizes the menopause leads to deep changes in the lipidogram in such women compromising the function of cardiovascular system.

Extensive epidemiological, clinical, and mechanistic studies have demonstrated higher risk of incidence of cardiovascular disease increases after menopause as a result, partially, to dyslipidemia and atherosclerosis [3,8]. Nevertheless, many evidences point out hormone replacement therapy (HRT) ameliorates either casuistic statistics as well as the severity of cardiovascular complications in post-menopausal women corroborating the protective role of estrogen in women [7,8]. Moreover, it has been shown that raise in serum lipids, such as LDL, is also accompanied by increase in body weight and fat body mass (FBM) in postmenopausal women. However, it is not clear yet whether the fat mass gain is the cause or the consequence of the increased serum lipid profile observed in those women. Recently, obesity has been linked to BC incidence in post-menopausal women [9]. Although metabolic syndrome is a multifactorial disease, recently both onto and epigenetic elements that influence in this syndrome have been discovered.

There has been shown that silent, low penetrance, genomic variations in key genes of dyslipidemia and atherosclerosis [3,8]. Particularly, SNPs PvuII c454-397T>C (NCBI ID: rs2234693) and XbaI c454-351A>G (NCBI ID: rs9340799) present on intron 1 of ESR1 gene have been associated to several estrogen-sensitive traits which include many pathological conditions such as abortion, breast cancer, endometriosis, myocardial infarction, and thromboembolism [6,9–17]. However, data concerning the role of these polymorphic elements into lipid profile in postmenopausal women are still inconclusive. In spite to clarify the association of PvuII and/or XbaI SNPs with lipid metabolism in postmenopausal women, we investigated the effect of the SNPs c454-397T>C and c454-351A>G (PvuII and XbaI, respectively) on serum total cholesterol (T-chol), total lipid (TL), low density lipoprotein cholesterol (LDL), high density lipoprotein cholesterol (HDL), and triglycerides (TG) serum levels aiming to correlate those elements to body mass index (BMI) and age of such patients. Also, following up several of these patients we could show that BC is associated to these SNPs.

## Materials and methods

### Study participants

Our group developed a comprehensive study focusing on polymorphisms in the estrogen receptor (ESR1) SNP in total of 499 participants [15]. From this *cohort*, the present study enrolled 419 healthy, multi ethnic (white, black and brown), highly miscigenated post-menopausal women aged between 49 and 91 years natural from Brazil. Women were considered post-menopausal if they had no menstruation for at least 12 months prior the blood collection and anamnesis. Any obvious physiological or pathological causes led to exclusion from the study at that time. Patients using any antidyslipidemic medication, showing liver or kidney insufficiency or under use of anti-HIV drugs were also excluded of study. All participants passed through a clinical anamnesis before they were included in this study. These patients were described at up 5 years measuring frequency of weight every year after genotyping (September, 2010). All patients received basically the same phamacological treatments for dyslipidemia. Adherence for body weight control was not massive for those patients who started the *cohort*. Our initial cohort had 419 patients but we end up with only 199 (Table 1) patients because chosed to use better controled patients we had at end point study (August, 2015). Moreover, every population were first of all statistically analyzed intragroup by One way ANOVA, resulting in p ≤ 0.001, proving to be a very homogeneous. As described in Statistical Analysis, different groups were compared using high strigent post hoc statistical test, Bonferroni’s test, and reach significance of p ≤ 0.05. Hence, we are confident that are results represent a very homogeneus as well as controled post-menopausal women population. The study was also approved by the Ethics Committee of the Federal University of Espírito Santo (UFES) and written consent was given by each participant (n° 018/2007).

**Table 1:** Population characteristics.

Genotype	Ethnicity	Age (years-old)	Weight (kg)	BMI (kg/m^2^)	N	BC
PP	White: 50.2%	64.8 ± 7.4	63.1 ± 10.5	26.1 ± 4.4	35	2
	Black: 2.4%					
	Brown: 47.4%					
Pp	White: 48.5%	64.7 ± 8.5	68.1 ± 14.8	30.2 ± 16.8	98	10
	Black: 6.7%					
	Brown: 44.8%					
pp	White: 52.6%	64.8 ± 8.7	67.0 ± 15.2	27.9 ± 5.9	60	11
	Black: 8.3%					
	Brown: 39.1%					
XX	White: 55.7%	64.6 ± 11.6	67.2 ± 14.2	29.3 ± 13.8	153	75
	Black: 5.8%					
	Brown: 38.5%					
Xx	White: 50.3%	64.8 ± 8.3	65.4 ± 11.6	26.5 ± 4.6	30	15
	Black: 6.5%					
	Brown: 42.7%					
Total	199	64.8 ± 8.3	66.8 ± 14.3	28.8 ± 12.6	199	149

Mean ± standard deviation values of age (years old); weight (Kg) and BMI (body mass index in each studied genotype). No significant differences between the genotypes studied were observed using T-test. Normal weight BMI 18.5–24.9 kg/m^2^; Overweight BMI 25–29.9 kg/m^2^; Obese BMI ≥ 30 kg/m^2^.

### DNA extraction and polymorphisms analysis

Genomic DNA samples were extracted from the specimens using a modified protocol of Goelz et al (1985) [18]. Briefly, blood specimens were incubated in 10 mg proteinase K diluted in 10% sodium dodecyl sulfate (SDS) solution at 60°C temperature for 2 hours. A solution of formaldehyde and chloroform (pH = 9) were added, and, after centrifugation (18,000xg for 2 minutes), supernatant was collected the genomic DNA precipitated with a solution 7.5 mM of ammonium acetate in ethanol absolute. Genomic DNA samples were finally washed 3x with 70% ethanol solution and ressuspend in ultra-pure DNAse/RNAse free water (all reagents were from Invitrogen, Carlsbad, CA, USA). PvuII c454-397T>C (NCBI ID: rs2234693) and XbaI c454-351A>G (NCBI ID: rs9340799) SNPs were analyzed by Polymerase Chain Reaction Restriction Fragment Lengths Polymorphism (PCR-RFLP) characterized by PAGE gel electrophoresis [15]. A 119kb DNA fragment that contains 2 polymorphic sites that were amplified using forward and reverse primers 5’ CTGTGTTGTCCATCACTTCATC 3’ and 5’ CCATTAGAGACCAATGCTCATC 3’, respectively. PCR reactions were performed through 30 cycles by the following steps: denaturation at 95°C for 60s; annealing at 52°C for 30s; and extension at 72°C for 30s. PCR products were digested with the restriction endonucleases PvuII and XbaI for 30 min at 37°C (Invitrogen, CA, USA). Digested DNA was then ran onto 10% polyacrylamide gel stained with silver nitrate a posteriori. Heterozygous Pp genotype exhibited 119, 78 and 41 bp lengths and heterozygous Xx genotype exhibited fragments 119, 88 and 31 bp lengths. Capital P or X represent the absence of restriction site while lower-case p or x indicate the presence of restriction site. Representative samples of all genotypes were confirmed by automatic sequence. For details see Silva et al. [17, 18].

### Serum lipid profile

Serum samples collected Total Cholesterol (T-chol), Total Lipids (TL), Low Density Lipoprotein Cholesterol (LDL-C), High Density Lipoprotein Cholesterol (HDL), and Triglycerides (TG) analysis. All analyses for accessing TL, T-chol, TG, HDL, and LDL serum concentration according to usual clinical analysis protocols (all obtained from Olympus, Brazil).

### Determination of Body Mass Index (BMI)

Anthropometric measurements were carried out during the initial screening. At all afore mentioned time points, body weight and standing height were measured in light clothing and with no shoes using a digital scale (Seca Alpha, model 770; Hamburg, Germany) with an accuracy of 1,000 ± 100 g and a commercial stadiometer (Leicester Height Measure; Invicta Plastics, Oadby, UK) to the nearest 0.5 cm. Body mass index (BMI) was calculated as weight (kg) divided by height squared (m^2^). Exact BMI, Fat mass, total body water, and lean mass was determined Dual-energy X-ray Absorptiometry (DXA, Faxitron, Switzerland, carried out at Centro de Diagnóstico e Pesquisa da Osteoporose do Espírito Santo–CEDOES (Vitória/ES, Brazil). Participants were categorized in two BMI groups according to World Health Organization (WHO) criteria [19]: Normal weight BMI 18.5–24.9 kg/m^2^; Overweight BMI 25–29.9 kg/m^2^; Obese BMI ≥ 30 kg/m^2^.

### Statistical analysis

Genotype distribution of the polymorphisms were determined by Hardy-Weinberg equilibrium using χ2 test. Haplotypes frequencies were estimated by using the software Arlequin 3.11 [18] based on the maximum likelihood model and the SEM algorithm. This software was also used to access the significance level of linkage disequilibrium resulting from the non-random association of the genotypes. All p-values lower than 0.05 were considered significant (p<0.05). Mean values for Total Lipids, Total Cholesterol, TG, HDL Cholesterol, and LDL Cholesterol were compared among all groups with each genotype using Student’s T-Test with Bonferroni *post hoc* correction and/or One Way ANOVA. To analyze the influence of age and BMI over the lipid profile in each genotype a linear regression analysis, followed by slopes comparison was calculated by Pearson´s coefficient where poor median, and good correlation, represents r^2^ ≤ 0.15, 0.16 ≥ r^2^≤ 0.45, and r^2^ ≥ 0.45, respectively. All statistical analysis were done using Origin Pro 8 software.

## Results

Recently, ESR1 SNPs c454-397T>C (also called PvuII–ID number rs2234693) and c454-351A>G (so called XbaI–ID number rs9340799) polymorphisms onto ESR1 gene have been shown to be associated to many diseases [6,9–14,17]. Herein this paper, it was investigated the role of such polymorphisms on the serum lipid profile of postmenopausal women associating it to body mass index and age. The studied population showed a distribution of the ESR1 PvuII alleles and genotypes: P = 43.6%; p = 56.4%; PP = 18.1%; Pp = 50.8%; pp = 31.1%. Analysis of frequency for ESR1 XbaI alleles and genotypes showed: X = 58.2; x = 41.8%; XX = 16.4%; Xx = 83.6%; xx = 0.0%. For PvuII SNP, such population showed at Hardy and Weinberg equilibrium (χ^2^ p = 0.435). However, the XbaI polymorphism equilibrium was not observed, due to the absence of xx genotype. Our group has previously shown that xx genotype is associated to high incidence of miscarriage and abortion, proving to be a rare genotype at least in Brazilian population [17]. It was also analyzed the combination of two genotypes, P and X, with the following frequencies observed: PPXX = 6.7%; PPXx = 11.2%; PpXX = 5.1%; PpXx = 43.2%; pp;XX = 4.0%; ppXx = 29.8%. The estimated haplotype frequency in the population was PX = 32.3%, px = 32.3%, Px = 9.8% and pX = 25.6%. As expected, a strong linkage disequilibrium between PvuII and XbaI polymorphisms was observed, probably due to the absence of the xx genotype (χ2 = 8.115; p< 0.01). The mean age of the whole population was 64.8 ± 8.4 years-old. The mean value for body weight was 66.9 ± 14.1kg and for BMI were 28.9 ± 12.3 kg/m^2^. Nevertheless, no differences among each genotype was observed in age and BMI (T-test p>0.05 –(Table 1).

**Fig 1:**
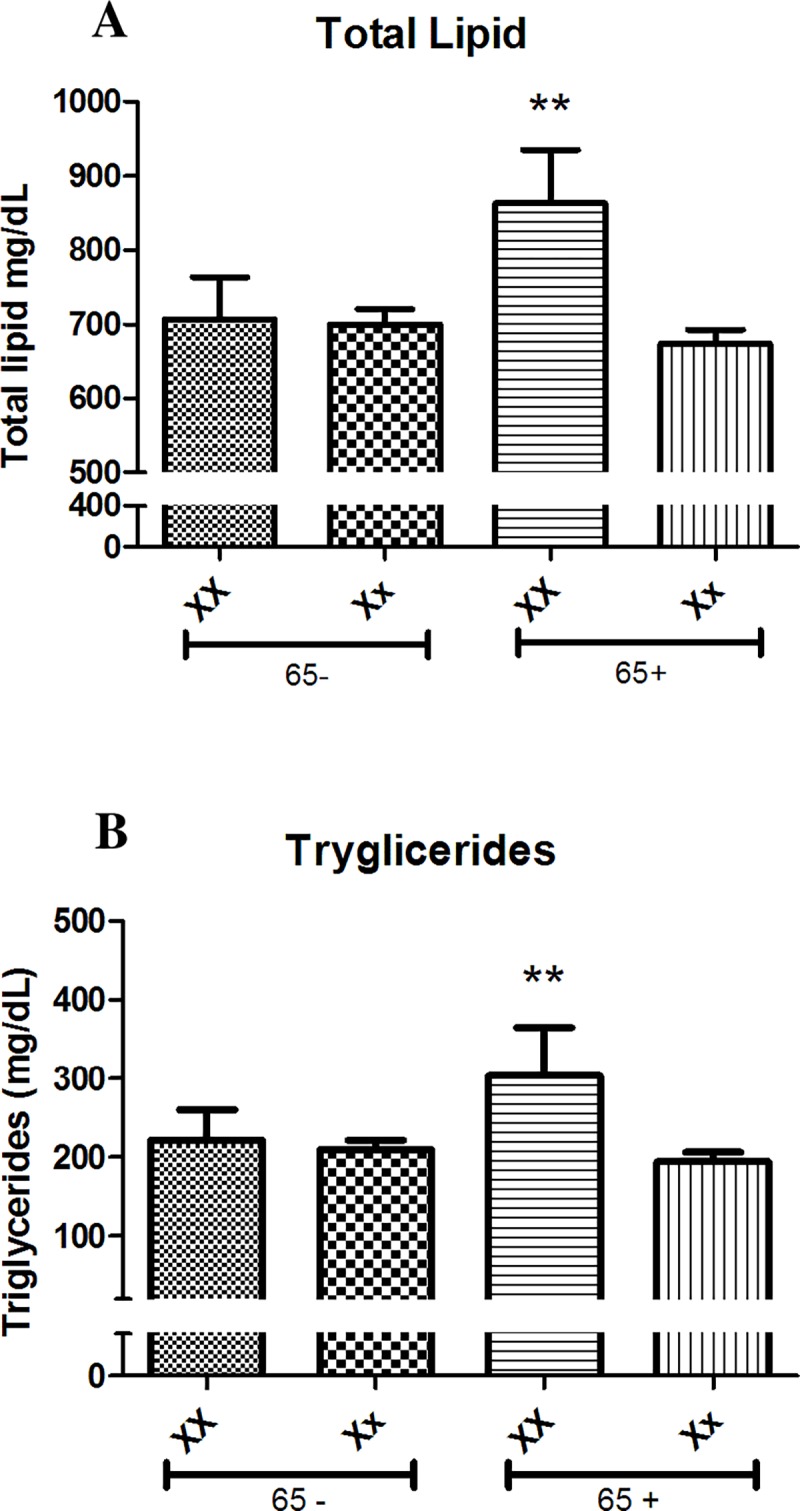
Lipid profile analyses of mean plasma levels Total Lipid (A) and Triglycerides (B) in women postmenopausal segregated by age (see 65-, women younger than 65 years-old; and 65+, women older 65 years-old)) and XbaI genotype (XX and xx). The effect of the genotypes was observed only in the older population ** T-test p<0.01 when compared to Xx women in the age group.

**Fig 2:**
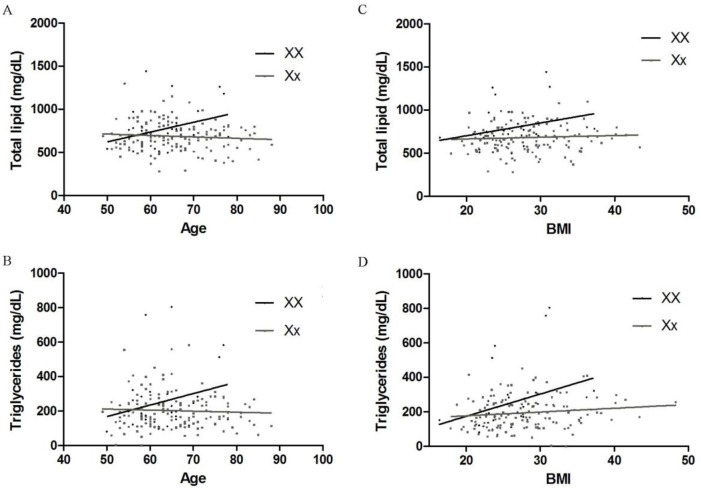
A: Linear regression graph comparing serum Total Lipids and age. Each lines slopes are significantly different (p = 0.015). Goodness of fit (r^2^): XX = 0.097; Xx = 0.007. B: Linear regression graph comparing serum Triglycerides and age. Each lines slopes are significantly different (p<0.05). Goodness of fit (r^2^): XX = 0.060; Xx = 0.002. C: Linear regression graph comparing serum Total Lipid and BMI. Each lines slopes are not significantly different (p>0.05). Goodness of fit (r^2^): XX = 0.072; Xx = 0.004. D: Linear regression graph comparing serum triglycerides and BMI. Each lines slopes are significantly different (p<0.05). Goodness of fit (r^2^): XX = 0.106; Xx = 0.017.

Analysis of the PvuII polymorphisms showed no effects over the mean values of serum Total cholesterol, TL, LDL, HDL, and TG. However, it was observed significant association of the XbaI SNPs over TG and TL whereas no differences between genotypes were seen for serum levels Total cholesterol, LDL, and HDL measurements (Table 2). Aiming to evaluate whether the role of genotype on the lipid profile is somehow dependent of the age of patients, we segregated our population in two groups with different characteristics–a former group younger as well as a later group older than 65 years-old. It was showed that the effect of XbaI polymorphism on the TL and TG serum concentrations was not present in women younger than 65 years-old. However, in women older 65 years-old, the influence of the genotype prevails, being significant between those XX and Xx genotypes (Fig 1A and 1B). Age´s effect was confirmed when XX and Xx patients were plotted according with their individual serum TL and TG concentrations, and a linear regression was performed (Fig 2A and 2B). It was observed that both parameters showed a positive correlation, with higher serum concentrations achieved in elder XX patients. Nonetheless, the Xx patients showed an opposite effect, with slightly decreasing values for both serum TL and TG concentrations. Line’s inclinations (measured by slope) were significantly different for both characteristics corroborating the association of age with dyslipidemic profile observed in older XX patients.

**Table 2:** Serum lipid profile for each genotype.

Genotype	Total Lipids	Triglycerides	Total Cholesterol	LDL	HDL
**PP**	726.4 ± 33.2	206.6 ± 23.6	231.6 ± 8.3	124.9 ± 7.2	65.3 ± 2.7
**Pp**	694.8 ± 18.6	206.9 ± 11.9	223.5 ± 4.9	120.6 ± 4.6	63.0 ± 1.8
**pp**	700.6 ± 25.5	212.7 ± 15.7	225.9 ± 7.1	118.7 ± 5.7	64.5 ± 2.3
**XX**	771.3 ± 46.0*	255.0 ± 34.3*	233.0 ± 9.4	118.0 ± 8.6	63.9 ± 3.0
**Xx**	687.0 ± 13.9	201.5 ± 8.6	223.6 ± 4.0	116.2 ± 3.5	64.6 ± 1.4

Data are expressed in mean values ± standard deviation of Total Lipids (mg/dL), Triglycerides (mg/dL), Total Cholesterol (mg/dL), LDL cholesterol (mg/dL), and HDL cholesterol (mg/dL) measured in each genotype.

* Statistically significant when compared XX to Xx genotype using Students T-Test corrected by post-hoc Bonferroni´s (p < 0.05).

**Fig 3:**
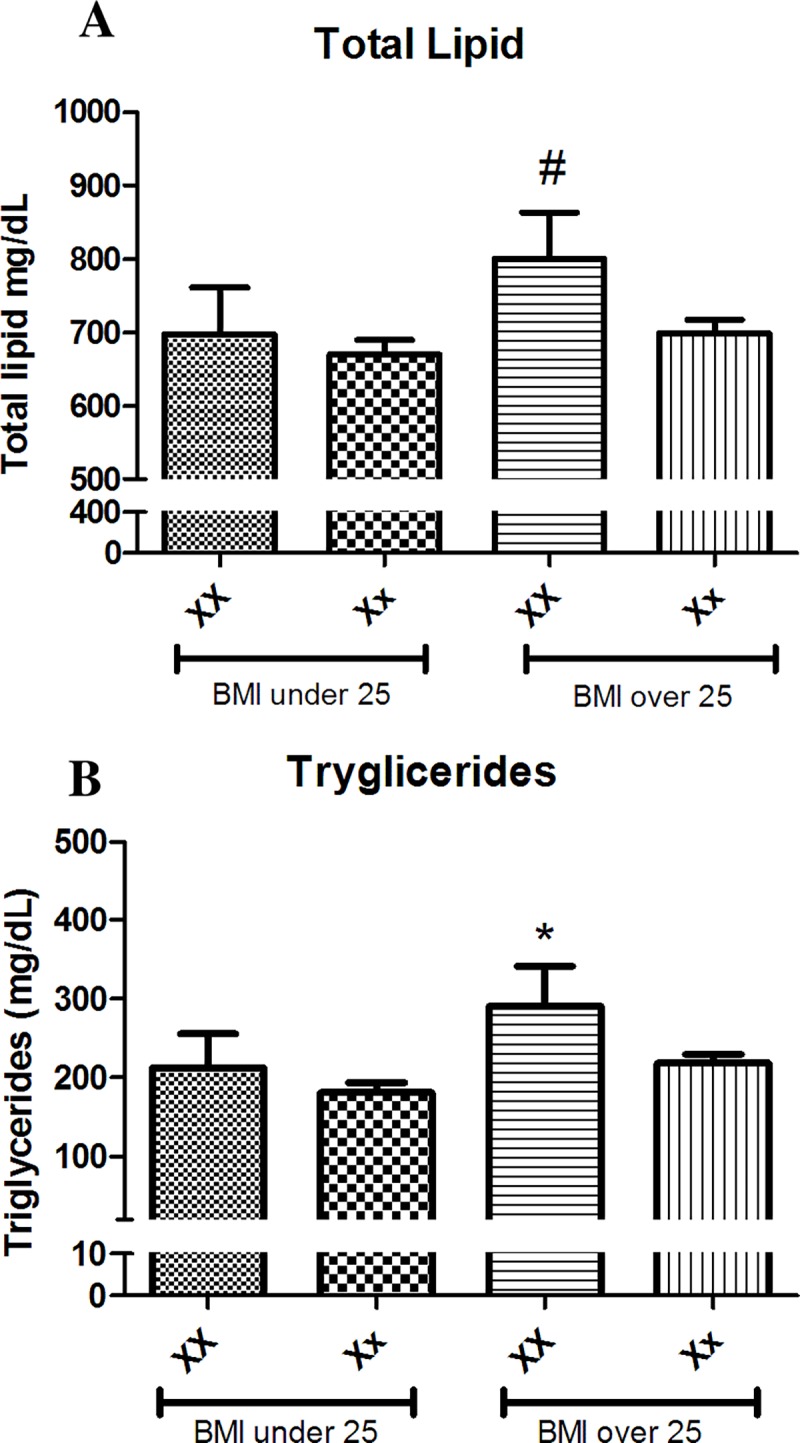
Profile lipids analyses of serum Total Lipids (A) and Triglycerides (B) mean values observed in women segregated by BMI and XbaI genotype. The effect of the genotypes was observed only in the over weighted population. ^≠^ T-Test p = 0.0527 comparing to Xx women in the same BMI group. * T-Test p < 0.05 comparing to Xx women in the same BMI group.

It also was investigated the dependency of BMI on the effect of the ESR1 polymorphisms on the TG and TL serum concentrations. To do so, the studied population were extractified in distinct groups: Normal weight BMI 18.5–24.9 kg/m^2^; Overweight BMI 25–29.9 kg/m^2^; Obese BMI ≥ 30 kg/m^2^. Interestingly, we observed that XX genotype only predicted a higher TL and TG serum concentration in women with higher BMI value (Fig 3A and 3B). To better evaluate whether the effect of high serum concentration of such parameters was due to the fat mass or the XX genotype all patient TL and TG concentrations were plotted (according to each genotype) along their BMI, and a linear regression was performed (Fig 2C and 2D). Analysis of curve genotype vs. lipid concentration revealed that XX patients exhibited an increased slope for serum TG concentration when compared to Xx patients (r = 0.32, good correlation). Moreover, Analysis of curve genotype vs. lipid concentration revealed that XX patients exhibited an increased slope for age of patient exhibited r = 0.40, good correlation). Results for TL were not statistically significant (see legend of Fig 2). Taken together, one may say that XX influences TG serum concentration in subject with higher BMI as well older age but not TL what may be due to the absence of effect in LDL, VLDL and HDL cholesterol. Patients carrying X allele had 75% of incidence of BC after 5 years of study (Table 1) which corroborated a previous study of our group [18].

## Discussion

Estrogen plays an important role in the regulation of lipids and lipoproteins metabolism. In humans, it has been shown that estrogen increases serum HDL cholesterol and triglycerides concentrations as well as decreases LDL cholesterol serum concentrations, mainly by modulating hepatic expression of genes involved in lipoprotein metabolism [6]. Moreover, estrogen may simultaneously up-regulate the expression of LDL receptor and apolipoprotein A-I (Apo A-I) in liver. On the other hand, estrogen down-regulates the hepatic lipase transcription, therefore, contributing to a health desired lipid profile [20–23]. Such pattern could, then, contribute for prevention of atheroma plaque formation with the blood vessels and, therefore, decreasing the risk of thromboembolism, heart attacks, and stroke. Many actions of the estrogen are mediated by the ESR1, the widespread isoform of this hormone receptor [1]. Besides its distribution, the concentration of this receptor reached into the target cells is as much relevant for the hormone action. Although the mechanisms of the ESR1 transcriptional regulation are poorly understood, recently a growing number of regulatory regions have been shown [24]. One of these transcript related regions is characterized as SNPs located onto the intron 1 of human ESR1 gene, identified as c454-397T>C and c454-351A>G, commonly called by PvuII and XbaI polymorphisms [6]. A possible functional mechanism attributed to PvuII and XbaI polymorphisms includes a change of ESR1 gene expression by altering the binding of its own transcription factors [2].

The present study analyzed the association of the SNPs c454-397T>C (also called PvuII—NCBI ID: rs2234693) and c454-351A>G (so called XbaI—NCBI ID: rs9340799) on ESR1 gene (NCBI ID: +133430)—which are believed to influence the expression of the ERalpha protein–on the serum lipid profile of postmenopausal women. It was found that the PvuII SNPs do not play any role on the serum T-chol, TL, LDL, HDL, and TG concentration. However, the XX genotype shows a deep effect on the TL and TG serum concentration in such women. Furthermore, those effects seem to be age and BMI dependent in postmenopausal women. Previous findings relying on the association of PvuII and XbaI polymorphisms on ESR1 gene with lipid profile are still controversial. Molvarec et al. [16] observed that serum T-chol concentrations were significantly higher in fertile women with the PP genotype whereas those women having XX genotype had significantly higher serum T-chol and LDL levels with no effects on other serum lipids. However, Almeida et al. [25] did not find an association between the two polymorphisms and serum lipid profile in a population of post-menopausal women with a mean age of 56 years-old. The data presented here, however, clearly shows that the higher serum concentrations of TL and TG are observed in patients older than 65 years-old but not in younger women (Fig 2A and 2B and Fig 3). Taken together, these data indicate that the homozygosis of X allele is an important factor on the TL and TG serum concentrations in elderly women. It is important to mention that there is a significant difference between the years followed the menopause observed in such groups (data not shown). This longer lack of exposition to estrogen may lead to the activation of ESR1 gene transcription among such women increasing the TL and TG production.

It is well documented that there is a gain of body weight, particularly an increase in visceral fat mass, after menopause and BC [18, 19, 26]. Our study also showed that, in overweight (BMI 25–29.9 kg/m^2^) and obese (BMI ≥ 30) post-menopausal women, the XX genotype is associated to higher levels of TL and TG, while no influence of this genotype was observed in the population considered to be normal or underweight (BMI 18.5–24.9 kg/m^2^). These data suggests that XX genotype is an important factor influencing the serum concentration of TL and TG mainly in overweight post-menopausal woman. When either total lipids or triglycerides are plotted by function of age or BMI significant increase is observed in both parameters in XX patients while no raise is seen for Xx (Fig 2). These data, however, corroborate that the association of XX with increasing serum concentrations of TL and TG is depending of both age and BMI. Although this association exists, we cannot state whether the higher triglycerides lead to obesity or the high fat mass only reflects in such hyperlipidemic profile.

In the present study, when both PvuII and XbaI polymorphic elements were associated to T-chol, LDL, HDL, and lipoproteins no association was found. Bagger et al. [27], in a study using 499 healthy Danish post-menopausal women, also did not observe any relationship between these polymorphisms and serum lipoprotein levels. Similarly, Matsubara et al. [28] did not find an association between the two polymorphisms (PvuII and XbaI) and serum lipid profile in a population of post-menopausal women as well. On the other hand, Herrington et al. [29]–in a study using familiar hypercholesterolemia patients–found that HDL levels to be slightly higher in women carrying PP genotype and Lu et al. [30] found the PP and XX genotype of the PvuII and XbaI polymorphisms were associated with lower serum HDL and ApoA-I levels whereas the XbaI xx genotype was related with lower serum ApoA-II levels. Taken together, these data may suggest that the effect of PvuII and XbaI polymorphisms on such serum lipids may be dependent of several other genes instead only the human ESR1 alone.

It is well known that PvuII and XbaI polymorphisms play an important role several pathological conditions such as osteoporosis, Alzheimer's disease, gynecological neoplasias, endometriosis, myocardial infarction, and thromboembolism [6,9–17]. The present study, however, aimed to evaluate how these polymorphisms affects lipid and lipoprotein serum concentrations in postmenopausal woman. Our finds showed no association between the PvuII polymorphism and serum concentration of TL, T-chol, LDL, HDL, and TG in post-menopausal woman. Nevertheless, XbaI polymorphisms showed a strong association between with higher serum levels of TL and TG, which seems to be also age and BMI dependent. Our results suggest that the XbaI polymorphism effects over the lipid profile in post-menopausal women may be related to the age, so that XX genotype predicts for dyslipidemic, obesity and some cases BC in older women.

One limitation of this study is the that one single gene polymorphisms is not enough to answer the actions of a complex disease such as dyslipidemia. Nonetheless to respond the all the causes of dyslipidemia and obesity we are also investigating other SNPs that have been previously asssociated to this illness, like SNPs on genes such as CYP19A1, ESR2 and PGR [31, 32].

## Abbreviations

ESR: estrogen receptor
ESR1: estrogen receptor alpha
PvuII: restriction enzyme PvuII
XbaI: restriction enzyme XbaI
T-chol: total cholesterol
TL: total lipid
LDL: low density lipoprotein cholesterol
HDL: high density lipoprotein cholesterol
TG: triglycerides
PCR-RFLP: polymerase chain reaction restriction fragment lengths polymorphism
HRT: hormone replacement therapy
SNP: single nucleotide polymorphisms
NCBI: National Center for Biotechnology Information
BMI: body mass index
SDS: sodium dodecyl sulfate
DXA: Dual-energy X-ray Absorptiometry
FBM: fat body mass
BC: breast cancer
